# Mr. James H. Chambers

**Published:** 1918-01

**Authors:** 


**Mr. James H. Chambers**, the founder and for more than 35 years the President of the Dios Chemical Co., St. Louis, Mo., died November 6.

